# Genomewide Identification and Characterization of the Genes Involved in the Flowering of Cotton

**DOI:** 10.3390/ijms23147940

**Published:** 2022-07-19

**Authors:** Xiao Li, Yuanlong Wu, Huabin Chi, Hengling Wei, Hantao Wang, Shuxun Yu

**Affiliations:** 1 National Key Laboratory of Crop Genetic Improvement, Huazhong Agricultural University, Wuhan 430000, China; lixiao_xiaoli@163.com (X.L.); wyl19880322@163.com (Y.W.); huabinc@foxmail.com (H.C.); 2 State Key Laboratory of Cotton Biology, Institute of Cotton Research of CAAS, Anyang 455000, China; henglingwei@163.com (H.W.); w.wanghantao@163.com (H.W.); 3 State Key Laboratory of Subtropical Silviculture, Zhejiang A & F University, Lin’an 311300, China

**Keywords:** upland cotton, genomewide, flowering-related genes, evolution, regulatory pathways, expression analysis

## Abstract

Flowering is a prerequisite for flowering plants to complete reproduction, and flowering time has an important effect on the high and stable yields of crops. However, there are limited reports on flowering-related genes at the genomic level in cotton. In this study, genomewide analysis of the evolutionary relationship of flowering-related genes in different cotton species shows that the numbers of flowering-related genes in the genomes of tetraploid cotton species *Gossypium hirsutum* and *Gossypium barbadense* were similar, and that these numbers were approximately twice as much as the number in diploid cotton species *Gossypium arboretum*. The classification of flowering-related genes shows that most of them belong to the photoperiod and circadian clock flowering pathway. The distribution of flowering-related genes on the chromosomes of the At and Dt subgenomes was similar, with no subgenomic preference detected. In addition, most of the flowering-related core genes in *Arabidopsis thaliana* had homologs in the cotton genome, but the copy numbers and expression patterns were disparate; moreover, flowering-related genes underwent purifying selection throughout the evolutionary and selection processes. Although the differentiation and reorganization of many key genes of the cotton flowering regulatory network occurred throughout the evolutionary and selection processes, most of them, especially those involved in the important flowering regulatory networks, have been relatively conserved and preferentially selected.

## 1. Introduction

The transition from vegetative growth to reproductive development is a major physiological change in the life cycle of higher plants. Flowering regulatory networks in model plant species such as *Arabidopsis thaliana* [1,2,3,4,5,6,7] and rice [1,8,9,10,11] were thoroughly studied. According to environmental factors affecting flowering, flowering regulatory networks can be divided into five types: those involving the photoperiod pathway [1,7,12,13,14,15], hormone pathway [16,17,18,19,20,21], vernalization pathway [2,22,23], aging pathway, and autonomous pathway [24]. Furthermore, each flowering pathway has core flowering regulatory genes [25] that undergo cross-talk among each other [25,26,27,28,29].

In the photoperiod pathway, day length is perceived by the leaves and induces a systemic signal called florigen that moves through the phloem sieve elements to the shoot apical meristem (SAM); the FLOWERING LOCUST (FT) protein is a major component of florigen [4,30] in different species, such as *Arabidopsis*, rice, and temperate cereals [8]. At the SAM of various plant species, such as *Arabidopsis*, rice, wheat, temperate crops, and cereals [1,2,4,30], the FT-FLOWERING LOCUS D (FD) complex is involved in the network associated with the SAM, and causes changes in the expression of floral meristem-identity genes, which reprogram the stem cell differentiation fate and change the fate of the axillary primordia to form floral primordia instead of leaf primordia [4,5,31]. *SUPPRESSOR OF OVEREXPRESSION OF CONSTANS 1* (*SOC1*), *FRUITFULL* (*FUL*), *AGAMOUS-LIKE 24* (*AGL24*), *LEAFY* (*LFY*) and *APETALA1* (*AP1*) involved in the initiation of flower primordia in the SAM are major meristem-identity genes in different species, such as *Arabidopsis*, rice, wheat, and various other cereals [1,2,4,30]. SQUAMOSA PROMOTER BINDING PROTEIN-LIKE 3 (SPL3) is a direct upstream activator of *LFY* and *AP1* [32], and it was investigated if *OsSPL14* can be regulated by OsmiR156 to define the ideal plant architecture in rice via facilitating branch primordia differentiation [33]. Moreover, in the leaves, there are varieties of photoreceptors that sense light signals; the blue light photoreceptors cryptochrome (CRYs) [34], the other blue light photoreceptors ZEITLUPE/FLAVIN-BINDING, KELCH REPEAT, and F-BOX/LOV KELCH PROTEIN 2 (ZTL/FKF1/LKP2) [15,35,36,37], and the red/far-red light photoreceptor phytochromes (PHYA to PHYE) [13,38,39,40] are three major photoreceptors that regulate flowering time in *Arabidopsis* [41,42]. Recently, significant progress has been achieved on light receptors functioning on flowering in species such as rice [43], wheat [1,2], soybean [44], longan [45], *Brassica juncea* [46] and tomato [47]. Additionally, under long-day conditions, *GIGANTEA* (*GI*) can also directly facilitate *FT* transcription in the leaves [48,49].

The hormone pathway refers to the requirement of the pathway of hormones, especially gibberellic acid (GA) [25], auxin (IAA), and brassinosteroids (BRs) [21], for normal flowering patterns. In flowering, BR signals strongly undergo cross-talk among the GA, IAA, and light signaling pathways [21]. DELLA proteins, which are the transcription inhibitors of GA signals, inhibit both *BRASSINAZOLE RESISTANT 1* (*BZR1*) and *PHYTOCHROME-INTERACTING FACTORs* (*PIFs*) to integrate the light, GA, and BR pathways [46]. Moreover, DELLA proteins are highly conserved among different species, including *Arabidopsis*, rice, maize, grape, wheat, barley, and *Brassica* [50,51]. BRs can repress the expression of *PHYB*/*D,* and activate the expression of *CONSTITUTIVELY PHOTOMORPHOGENIC1* (*COP1*) and *SUPPRESSOR OF PHYTOCHROME A 1* (*SPA1*) to regulate photomorphogenesis in *Arabidopsis* [21]. However, although BRs were first discovered in 1979 in extracts of *Brassica napus* pollen [51], the basic signaling pathway appears to be highly conserved in higher plants, the current BR network is mainly based on *Arabidopsis*, and the BR-signaling network in different plant species remains unclear [21]. 

Major floral initiation repressor FLOWERING LOCUS C (FLC), which is the core component of the vernalization pathway, can perceive changes in environmental temperature, regulate the expression of *FT* in leaves both in *Arabidopsis* and other species such as *Aquilegia* and *Brassica napus* [52,53,54,55], and respond to FT signals by directly repressing the expression of *SOC1* and preventing the upregulation of *FD* in the SAM [22,52]. The autonomous pathway primarily facilitates flowering by downregulating *FLC* through RNA processing and epigenetic regulation. The crucial components involved in this pathway include FLOWERING LOCUS CA (FCA), FLOWERING LOCUS KH DOMAIN (FLK), FLOWERING LOCUS PA (FPA), FLOWERING LOCUS D (FLD), FLOWERING LOCUS VE (FVE), LUMINIDEPENDENS (LD), and FLOWERING LOCUS Y (FY) [24,25,54,56]. Most of the abovementioned genes are major components of autonomous pathways in various plant species, such as rice [57], canola [58], *Doritaenopsis* [59], and soybean [60]. 

These flowering pathways are not independent of each other; there is a complex link between them [25]. Flower repressor *TEMPRANILLO* (*TEM*) plays a pivotal role in the direct repression of *FT* by directly repressing the expression of the GA biosynthesis-related genes *GA_3_*-*oxidase 1* and *2* (*GA3OX1* and *GA3OX2*) to link the photoperiod and hormone pathways for flowering in *A. thaliana* [28,61]. Additionally, in soybean, BR can reduce the transcript level of *GmRAV,* the family to which *TEMs* belong [62]. Moreover, the balance between CONSTANS (CO) and TEM proteins hastens *FT* expression to trigger flowering [26]. GA biosynthesis-inhibiting gene *SHORT VEGETATIVE PHASE* (*SVP*) is involved in the control of flowering in *Arabidopsis* [29,63]. It was also investigated in other plant specises such as wheat [64], and temperate fruit trees including apple [65], *Prunus mume* (mei) [66], sweet cherries [67], and kiwifruit [68]. 

Cotton plants, which are members of the *Malvaceae* family and *Gossypium* genus, are considered to be a model species for the study of plant polyploidy [69]. Since most of the planted varieties are allotetraploids, the cotton genome is more complex, coupled with complex traits and a long growth period, and studies on flowering are key to finding a way to regulate flowering time to breed early-maturity cotton. Cotton flowering is positively correlated with the differentiation of flower primordia. However, cotton flower primordia differentiation is driven by axillary bud primordia, which is governed by SAM differentiation. Axillary bud primordia differentiate into leaf primordia during early growth and development; however, they differentiate into flower primordia during later growth and differentiation, marking the beginning of the cotton transition from vegetative growth to reproductive development.

Previous genomewide association study (GWAS) results showed that flowering time, the length of the period from the first flower bloom to the first boll opening, the length of the whole growth period, the yield percentage before frost, and the plant height and height of the first branch node were early-maturity traits, and some important flowering-related alleles have been identified, such as *GhSPY*, *GhZTL*, *GhELF6*, *GhSVP*, *GhELF4*, *GhGA2OX6,* and *GhPHYA* [69]. Furthermore, the GhCAL protein can form heterodimers with GhAP1 and GhAGL6 to regulate these two genes’ expression and facilitate cotton flowering [70]. Coincidentally, these flowering-related genes are highly homologous to those in *A. thaliana*, suggesting that the key flowering mechanism was relatively conserved during the evolution and domestication of plant species. However, how strong selection pressure alters the genome of cotton, particularly the genetic components underlying the adaptation to environmental changes and improved early maturity, remains unknown.

In comparison with extensive studies of the flowering mechanisms in model plant species *A. thaliana*, the flowering mechanisms in cotton remain unknown. In this study, to better understand the flowering mechanism of cotton, flowering-related candidate genes were identified with genomewide identification-based techniques. A detailed analysis of the flowering-related genes, including their functional classification, chromosomal locations, phylogenetic relationships, conserved motifs, and expression patterns, was performed. Moreover, the nonsynonymous substitution rate (Ka), synonymous substitution rate (Ks), and ω (Ka/Ks) ratio were calculated to determine the divergence time and selection pressure of homologous gene pairs. This systematic analysis of flowering-related genes in cotton provides a basis for further studies on the flowering mechanism of cotton.

## 2. Results

### 2.1. Comparison of Flowering-Related Genes from Arabidopsis thaliana, Gossypium hirsutum, Gossypium barbadense, and Gossypium arboretum

With the development and improvement of the *A. thaliana* flowering-related genomic network, 306 flowering time-related genes have been found, and information about their evolution and an interactive database, FLOR-ID, can be found at http://www.flor-id.org (accessed on 3 November 2021) [71]. On the basis of the protein sequences of the 306 flowering-related genes in *A. thaliana*, we investigated the flowering-related homologous genes in *G. hirsutum* and *G. barbadense*. In total, 636 and 673 flowering-related genes were identified in *G. hirsutum* (Table S1) and *G. barbadense* (Table S2), respectively, and more than half of the *A. thaliana* flowering-related genes had putative *G. hirsutum* (176 out of 306 genes) and *G. barbadense* (174 out of 306 genes) homologs. As in the *G. barbadense* genome, homologs in the *G. hirsutum* genome were lacking for 130 genes, and most of these genes (120 of 130 genes) have a single functional effect on flowering (Tables S3 and S4). Furthermore, 350 flowering-related genes in *G. arboreum* were identified (Table S5), and more than half of the flowering-related genes in *A. thaliana* had putative homologs in *G. arboretum* (173 out of 306 genes; Table S5); in addition, 133 genes have been lost (Table S6).

**Figure 1:**
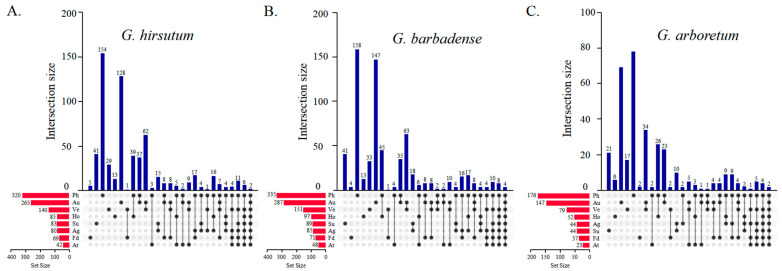
Identification and classification of flowering-related genes in *G. hirsutum*, *G. barbadense*, and *G. arboretum*. (**A**) *G. hirsutum*. (**B**) *G. barbadense*. (**C**) *G. arboretum*. The histogram on the left represents the number of flowering-related genes of each flowering-regulated pathway, a single black point in the middle matrix represents the number of genes specific to a pathway, the lines between the black points represent the intersection of the different pathways, and the vertical blue histogram represents the corresponding intersecting gene numbers. Ph: photoperiodism pathway; Au: autonomous pathway; Ho: hormone pathway; Ve: vernalization; Ag: aging pathway; Su: sugar signal; Fd: flower development and apical meristem response pathway; At: ambient temperature pathway.

To explore the flowering mechanism among the cultivated cotton species (*G. hirsutum*, *G. arboretum,* and *G. barbadense*), the putative flowering-related genes were classified into the eight following gene sets according to the classification of *A. thaliana* genes: those involved in the photoperiod pathway, circadian clock, and light signaling (Ph); sugar (Su); vernalization (Ve); the autonomous pathway (Au); hormone signaling and metabolism (Ho); the aging pathway (Ag); flower development and the apical meristem response pathway (Fd); and the ambient temperature pathway (At) [71]. Interestingly, most of the lost (53) genes belonged to the photoperiod pathway in *G. hirsutum*. There were 320 genes involved in the photoperiod pathway, and there were too many overlapping genes between the photoperiod and vernalization (62 genes), and hormone (39) pathways. The fewest common genes were detected between the photoperiod and ambient temperature pathways (three genes) (Figure 1A), and similar results were found in *G. barbadense* (Figure 1B).

The number of flowering-related genes in *G. hirsutum* (636 genes) and *G. barbadense* (673 genes) was approximately twice that in *G. arboretum* (350 genes, Figure 1 and Figure S1). Moreover, there were no significant differences in the number of flowering-related genes between *G. hirsutum* (636 genes) and *G. barbadense* (673 genes). By classifying the putative flowering-related genes in *G. hirsutum*, *G. barbadense,* and *G. arboretum* into the eight gene sets according to the *A. thaliana* dataset, we found that most of the genes participate in the photoperiod pathway (Figure S1). Additionally, there was no significant difference in the number of *G. hirsutum* and *G. barbadense* genes associated with each gene set (Figure S1). 

Additionally, the hormone signaling gene sets (16.07% were lost) or meristem response and development gene sets (20% were lost) were preferentially retained instead of the other pathway genes. However, more than 30% of the light signaling (38.78%), vernalization (30.86%), and aging (47.5%) genes were lost (Table S3). 

### 2.2. Chromosomal Localization and Duplication Analysis of Flowering-Related Genes in G. hirsutum and G. barbadense 

**Figure 2:**
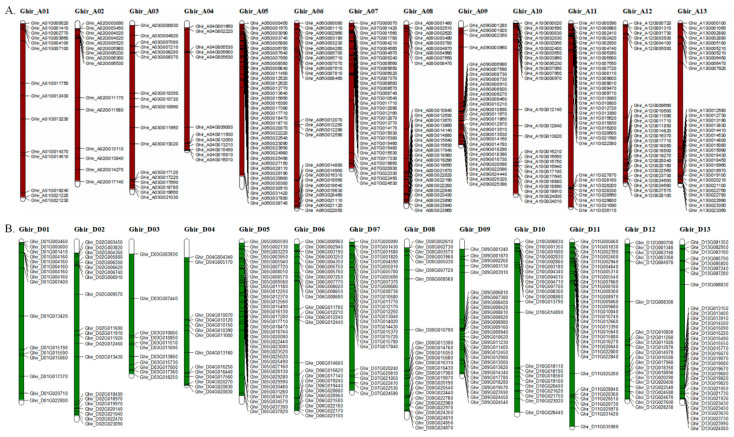
Distribution of flowering-related genes on *G. hirsutum* chromosomes. (**A**) Brownish bars: pseudochromosomes of the At subgenome; green bars: pseudochromosomes of the Dt subgenome. (**B**) Black lines on brownish and green bars indicate the locations of flowering-related genes on the pseudochromosomes. Values corresponding to the scales on the black vertical line indicate the physical distances.

The 636 and 673 flowering-related genes in *G. hirsutum* (Figure 2 and Table S7) and *G. barbadense* (Figure S2 and Table S8), respectively, were mapped onto pseudomolecular chromosomes. These genes exhibited equal genomic distribution, with 324 (*G. hirsutum*) and 329 (*G. barbadense*) genes localized on the At subgenome, and 310 (*G. hirsutum*) and 329 (*G. barbadense*) genes localized on the Dt subgenome. However, the chromosomal distribution of these genes was uneven. For the At subgenome, 37 (*G. hirsutum*) and 34 (*G. barbadense*) genes were localized on chromosome A05, with most located on the top half, representing 11.42% (*G. hirsutum*) and 10.33% (*G. barbadense*) of the flowering-related genes in the At subgenome (Figure 2A and Figure S2A). Only 12 flowering-related genes were detected on chromosomes A04 (3.70%, *G. hirsutum*) and A02 (3.65%, *G. barbadense*), with most distributed at both ends (Figure 2A and Figure S2A). In addition, 34 (10.97%, *G. hirsutum*) and 33 (10.03%, *G. barbadense*) genes were located on chromosome D05, with most located on the top half. Only 11 genes (3.55%) were detected on chromosome D03 of *G. hirsutum*, and 12 genes (3.65%) were detected on D01 and D04 of *G. barbadense*, with most located on the bottom half (Figure 2B and Figure S2B). Interestingly, most of the other genes were distributed near the ends of chromosomes (Figure 2 and Figure S2).

### 2.3. Floral Pathway Integrators

**Figure 3:**
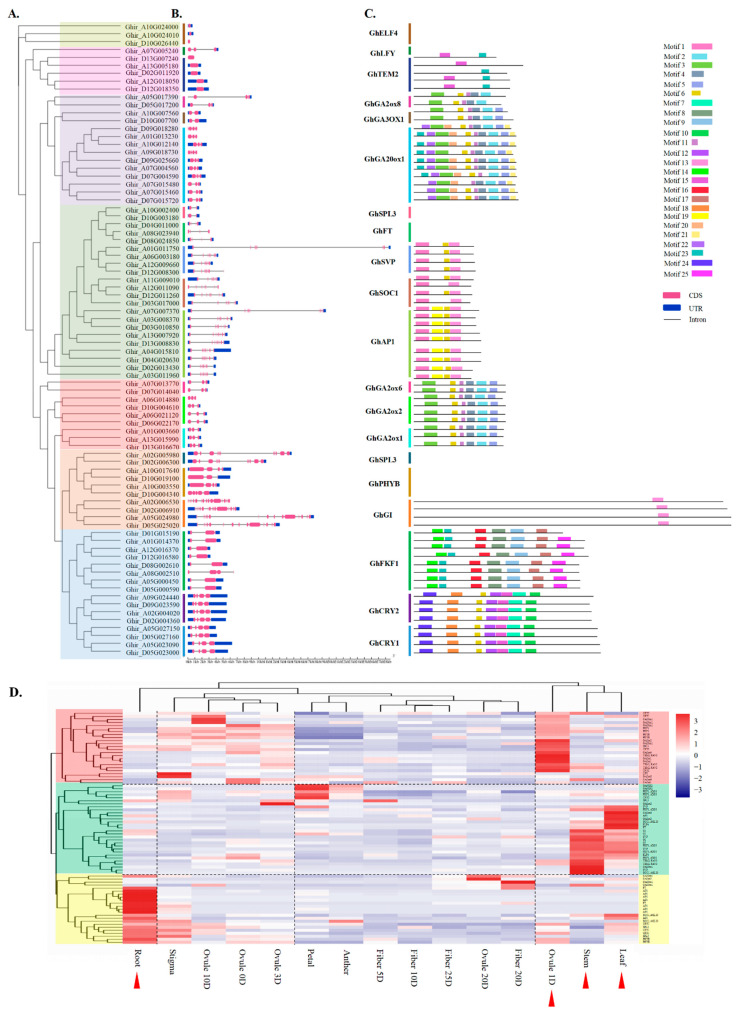
Distribution of the conserved motifs and exon–introns of photoperiod pathway flowering-related genes in *G. hirsutum*. (**A**) Phylogenetic tree of photoperiod pathway flowering-related genes in *G. hirsutum* constructed via the MEGA 6.0 program with the maximum likelihood (ML) method and 1000 bootstrap replicates. (**B**) Exon–intron structure of *G. hirsutum* flowering-related genes involved in the Ph pathway, as revealed by TBtools software. (**C**) Distribution of the conserved motifs of the flowering-related genes; the conserved motifs are indicated by the colored boxes. (**D**) Expression profiles of flowering-related genes. The expression profiles of flowering-related genes were detected in 15 different tissues: root, stigma, ovule 0D, ovule 1D, ovule 3D, ovule 10D, ovule 20D, petal, anther, fiber 5D, fiber 10D, fiber 20D, fiber 25D, stem and leaf tissues. Red arrows indicate the tissues in which the genes were highly expressed. D, day.

In flowering, endogenous elements always undergo cross-talk with exogenous factors through certain integrators [4]. The functional classification of flowering-related genes showed that systemic signal *FT* [1,4,8,28], which is called *GhFT* or *TWIN SISTER OF FT* (*GhTSF*) in *G. hirsutum*, functions in all eight flowering pathways, namely, Ph, Ho, Ag, At, Ve, Fd, Au, and Su [1,2,4,7,8] (Figure 3). In addition, floral pathway integrator SOC1 [72,73], and floral meristem-identity genes *AP1* [2,74,75], *LFY* [76,77,78] and *SVP* [79] (Figures S3 and S4) are found in *G. hirsutum*. *GhSOC1*, also named *GhAGL20* and *GhLFY*, participates in the Ph, Ho, Ag, At, Ve, Fd and Su pathways. *GhAP1*, also called *GhAGL8*, is regulated by the Ph, Fd, Ag, At, Su and Ve flowering pathways, and *GhSVP,* also called *GhAGL24*, works in the At, Ag, Ph, Fd and Ve flowering pathways in *G. hirsutum* (Figure S3).

Furthermore, phylogenetic analysis revealed that floral pathway integrators *GhFT*, *GhSOC1*, *GhSVP* and *GhAP1* have a close genetic relationship (Figure 3A), which is consistent with the findings of our exon–intron structural analysis; 3 identified *GhFT* homologs contained 4 exons, 4 *GhSVP* homologs contained 7 exons, and 9 *GhAP1* homologs contained 7 to 8 exons (Figure 3B).

To systematically determine the conserved motifs of the flowering-related genes involved in the Ph pathway, the distribution of conserved motifs of these genes was estimated by using the online MEME server in conjunction with 25 putative conserved motifs (Figure S5). Results show that the length of the conserved motifs ranged from 19 to 50 amino acids, and the number of conserved motifs within each flowering-related gene involved in the Ph pathway ranged from 0 to 10 (Figure 3C,D), showing that all *GhSVP*, *GhAP1* and *GhSOC1* homologs contained three conserved motifs. Interestingly, the three *GhFT* homologs contained no conserved motifs with other Ph pathway flowering-related genes (Figure 3C).

### 2.4. Flowering-Related Genes Involved in the Photoperiod Pathway, Circadian Clock, and Light Signalling

Light is indispensable for plant life, and the perception of the light environment dictates seed germination, photomorphogenesis, phototropism, shade avoidance, and flowering [61]. In this study, 320 genes in *G. hirsutum* homologous to 174 genes related to the photoperiod pathway, circadian clock, and light signaling in *A. thaliana* were identified (Figure 1 and Table S1). To explore the evolution and genomic structure of Ph pathway-related genes, the phylogenetic analysis and intron diagrams of Ph pathway flowering-related genes were generated on the basis of their sequences (Figure 3A,B). The findings show that *GhSPL3*, *GhGI*, and photoreceptors *GhFKF1*, *GhCRY1/2,* and *GhPHYB* are closely genetically related (Figure 3A). The exon number of each gene widely ranged from 1 to 15 exons. Significantly, 3 *GhELF4* genes were identified, and all of them contained only 1 exon, 2 *GhSPL3* copies had 10 exons, and 4 *GhPHYB* copies had 3 to 5 exons. Four *GhGIs* were identified and contained 14 to 15 exons (Figure 3B). *FKF1* belongs to a family of F-box proteins of which *ZTL* and *LKP2* are members, and all of them comprise light, oxygen, or voltage (LOV) domains [35]. Like in *G. barbadense*, there are eight copies of each of the genes *GhZTL*, *GhLKP2,* and *GhFKF1* in *G. hirsutum* (Table S2). Moreover, seven *GhFKF1* genes contained two exons (Figure 3B). Blue light photoreceptors CRY1 and CRY2, which monitor light signals to regulate plant flowering [34,80], were identified in *G. hirsutum* (Table S1), and four *GhCRY1* and *GhCRY2* contained four exons (Figure 3B). 

Furthermore, most GA biosynthesis homologs, such as *GA2ox*, *GA3ox*, and *GA20ox* [81], were retained (Figure 3); they were simultaneously induced in response to light signaling and contained six conserved motifs in *G. hirsutum* (Figure 3C and Table S1). Our conserved motif analysis showed that genes *GhELF4*, *GhLFY*, *GhSPL3,* and *GhPHYB* had no conserved motifs. However, the five *GhTEM2* genes had two conserved motifs, the *GhGI* genes contained one conserved motif, the *GhFKF1* genes had eight conserved motifs, and the *GhCRYs* had seven conserved motifs (Figure 3C).

### 2.5. Expression Analysis of Ph Pathway Genes in G. hirsutum

To investigate the divergence in the expression levels of homologous genes, and their participation in the photoperiod and circadian clock pathways, we analyzed the expression patterns of these supposed flowering-related genes in *G. hirsutum*, including the root, stem, leaf, petal, anther, stigma, ovule 0D (Day), ovule 1D, ovule 3D, ovule 10D, ovule 20D, fiber 5D, fiber 10D, fiber 20D and fiber 25D tissues (Figure 3D and Table S9). The results showed that most of the expressed genes were in the roots, petals, stems and leaves (Figure 3D and Table S9). Four *GhCRY1/2*, four *GhGA20ox1*, two *GhFKF1*, two *GhPHYB*, three *GhTEM2* and one *GhSPL3* were specifically or preferentially expressed in ovule 1D tissue. Four *GhSVP*, four *GhFKF1*, four *GhGI*, two *GhSOC1*, two *GhGA2ox*, two *GhELF4*, one *GhAP1,* and one *GhFT* were specifically or preferentially expressed in the stems and leaves. Eight *GhAP1*, three *GhCRY1*, two *GhSOC1*, two *GhPHYB*, two *GhSPL3,* and two *GhFT* genes were specifically or preferentially expressed in the roots or leaves. Only one copy of *GhLFY* was found in *G. hirsutum,* and it was preferentially expressed in the roots (Figure 3D).

### 2.6. Hormone-Pathway-Related Flowering-Related Genes

**Figure 4:**
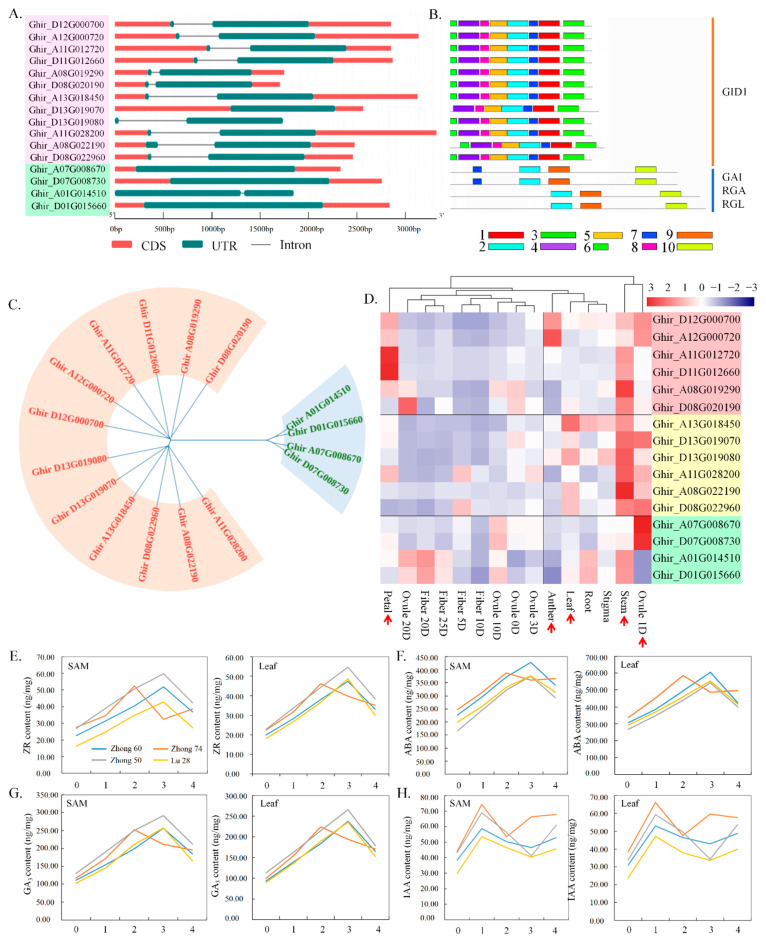
Distribution of conserved motifs and exon–intron structure, and expression level analysis of Ho pathway flowering-related genes. (**A**) Exon–intron structure of *G. hirsutum* flowering-related genes involved in the Ho pathway, as revealed by TBtools software. (**B**) Distribution of conserved motifs of flowering-related genes; conserved motifs are indicated by colored boxes. (**C**) Unrooted phylogenetic tree of Ho pathway flowering-related genes in *G. hirsutum* according to the MEGA 6.0 program with the maximum likelihood (ML) method and 1000 bootstrap replicates. (**D**) Tissue expression patterns of key genes involved in the *G. hirsutum* photoperiod pathway. The abscissa represents different tissues, and the rightmost ordinate represents the gene ID and name. Red arrows indicate the tissues in which the genes were highly expressed. (**E**–**H**) Changes in hormone contents ((**E**) ZR; (**F**) ABA; (**G**) GA3; (**H**) IAA) in the SAM and leaves of cotton at different true-leaf flattening stages (first true-leaf stage 1TLS, second true-leaf stage 2TLS, third true-leaf stage 3TLS, fourth true-leaf stage 4TLS). The abscissa represents the development stages for cotton. o, cotyledon period; 1, the first true-leaf stage; 2, the second true-leaf stage; 3, the third true-leaf stage; 4, the fourth true-leaf stage. The vertical axis represents hormone content. Two early-maturing cultivars: Chinese Cotton Research Institute 50 (CCRI50, also named Zhong50) and Chinese Cotton Research Institute 74 (CCRI74, also named Zhong74); two late-maturing cultivars: Chinese Cotton Research Institute 60 (CCRI60, also named Zhong60) and Luyanmian28 (Lu28). SAM, shoot apical meristem.

In *A. thaliana*, by controlling the spatial expression of floral regulatory genes throughout the plant independent of light signals, GA signals promote the initiation of floral primordia [27]. 85 genes likely related to the hormone pathway were identified in *G. hirsutum* genome. GA is perceived by its receptor, GID1, and there are 12 *GhGID1s* in *G. hirsutum* (Figure 4A–C and Table S1). CID1s can bind to bioactive GA through the conformational changes, which then facilitates the interaction between CID1 and DELLA proteins, which are major flowering repressors [82,83,84]. There are five DELLA family genes, namely, *GIBBERELLIC ACID INSENSITIVE* (*GAI*), *REPRESSOR OF ga13* (*RGA*), *RGA-LIKE1* (*RGL1*), *RGL2* and *RGL3*, in the *A. thaliana* genome [85,86]. We found four *GhGAIs*, namely, *GhRGA*, *GhRGL1*, *GhRGL2* and *GhRGL3* in *G. hirsutum* (Figure 4A–C and Table S1).

To further study the structural features of these genes, the exon–intron structure was analyzed, the results of which show that the 12 *GhGID1s* possessed 2 exons, and the 4 *GhGAI* only had 1 exon (Figure 4A). To visualize the conserved residues, the Ho pathway flowering-related gene motifs were analyzed with 10 putative conserved motifs (Figure S6), the results of which show that the *GhCID1* genes have eight conserved motifs, two *GhGAI* genes have four conserved motifs, and one *GhRGA* and one *GhRGL* have three conserved motifs (Figure 4B). Phylogenetic analysis revealed that the *GhGID1s* and *GhGAI* genes are closely related to their homologs (Figure 4C). The expression pattern analysis of 15 different cotton tissues showed that *GhGAI* has divergent expression patterns among their different copies coupled with sequence similarity, five *GhGAI* genes were highly expressed in petals, and six other genes were expressed in the leaf, stem, and ovule 0D tissues. Two *GhGAI* genes were highly expressed in ovule 0D; in contrast, two other genes showed nearly no expression in ovule 0D (Figure 4D and Table S9). 

We detected content changes in hormones zeatin riboside (ZR), abscisic acid (ABA), GA, and IAA in the SAM and leaves of two early-maturing cultivars Zhong50 and Zhong74; two late-maturing cultivars Zhong60 and Lu28 at different true-leaf flattening stages (first true-leaf stage 1TLS, second true-leaf stage 2TLS, third true-leaf stage 3TLS, and fourth true-leaf stage 4TLS). Results show that, during the development of the SAMs in cotton, the contents of hormones ZR, ABA, and GA are maximized; conversely, hormone IAA reached its minimal value during the second to third true-leaf stages (Figure 4E–H). Moreover, the balance between various endogenous hormones peaked at 2 TLS in cotton cultivars Zhong74, and at 3 TLS in Zhong50, Zhong60 and Lu28 (Figure S7). 

### 2.7. Selection Pressure on Flowering Pathway Gene Sets

**Figure 5:**
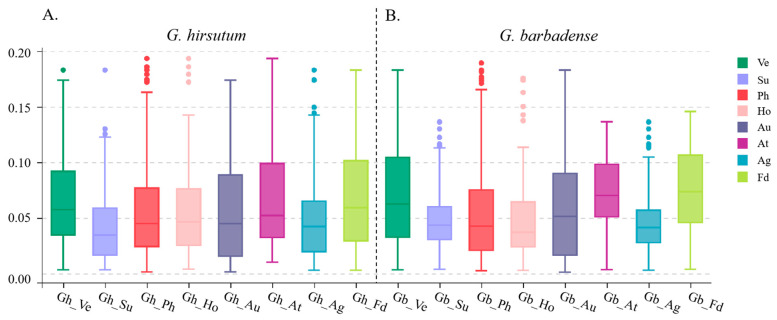
Direction and magnitude of natural selection acting on different flowering-related gene sets. Ka/Ks values of flowering-related genes in *G. hirsutum* (**A**) and *G. barbadense* (**B**). Quantile boxplots showing the distribution of Ka/Ks values for homologous gene pairs. Horizontal bars in each box indicate median values. Upper and lower bars correspond to the upper and lower adjacent values 1.5 times past the interquartile range. The outliers are plotted as discrete dots. Ve, vernalization; Su, sugar; Ph, photoperiod pathway, circadian clock, and light signaling; Ho, hormone signaling and metabolism; Au, autonomous pathways; At, ambient temperature pathway; Ag, aging pathway; Fd, flower development and apical meristem response pathway.

To investigate the evolution process, the nonsynonymous/synonymous substitution ratio (Ka/Ks), which is related to the evolutionary selection patterns of the corresponding genome [87], of flowering-related gene orthologous pairs between the *G. hirsutum* and *A. thaliana* genomes as well as between the *G. barbadense* and *A. thaliana* genomes were computed to determine the selection pressure. The calculation of the selection pressure showed that the mean Ka/Ks ratios between *A. thaliana* and *G. hirsutum* of different flowering-related gene sets ranged from 0.001 to 0.200 (Figure 5, Tables S10 and S11). In particular, genes involved in flower development and the apical meristem response pathway appear to have been subjected to less negative selection pressure than the other pathway genes were. Moreover, the range of the selection pressure variation was largest in Ph pathway genes during the evolution of cotton (Figure 5A,B, Tables S10 and S11).

## 3. Discussion

**Figure 6:**
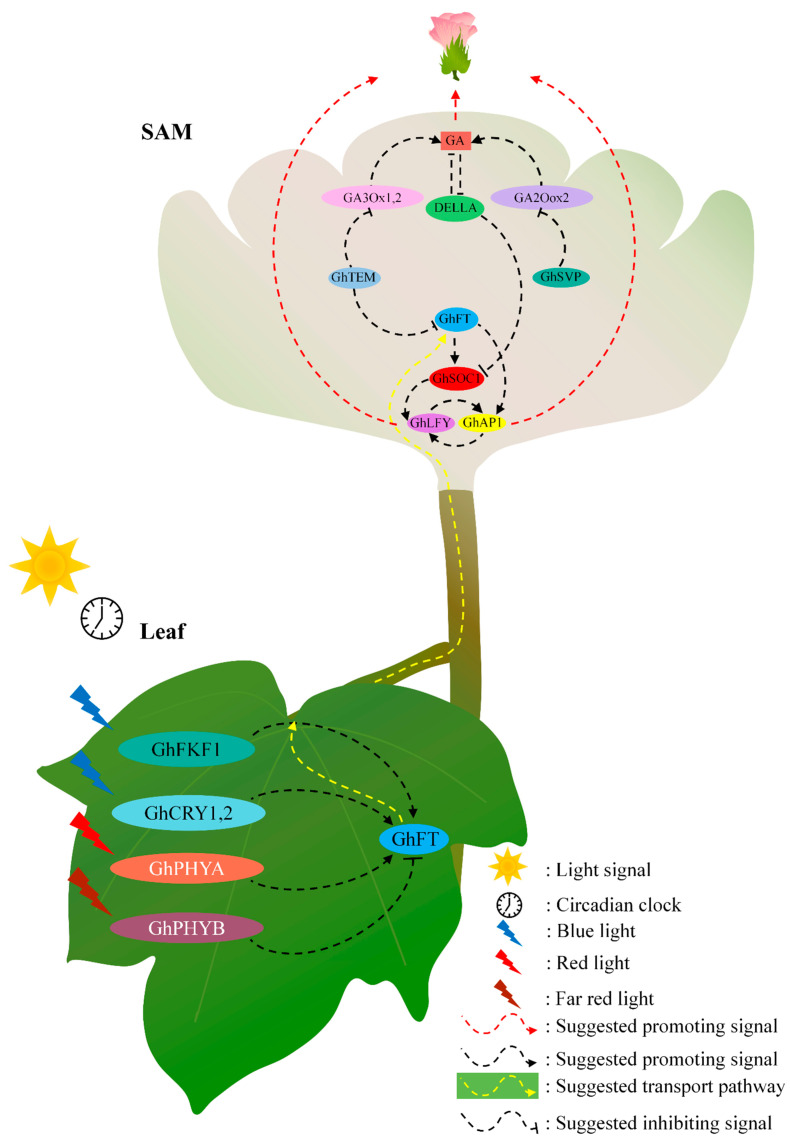
Possible regulatory model of the multiple feedbacks and inputs during the regulation of cotton flowering in the leaves and SAM.

Compared with the *A. thaliana* flowering mechanism, which has been comprehensively described, the exploration of cotton flowering mechanisms is still deficient. Cotton species *G. hirsutum*, *G. barbadense,* and *G. arboretum* were sequenced, and the release of genomic data makes it greatly convenient for studying the genes and functions of important agronomic traits [88]. Here, a bioinformatics approach was used to analyze the *G. hirsutum* and *G. barbadense* genes that potentially participate in flowering, and implied a possible regulatory model of the multiple feedbacks and inputs during the regulation of cotton flowering in the leaf and meristem (Figure 6).

In this study, the number of flowering-related genes identified in *G. hirsutum* was nearly equal to that in *G. barbadense* and twice that in *G. arboretum*, which is consistent with the results that *G. hirsutum* and *G. barbadense*, both of which are allotetraploid cotton species, were formed by the two closest diploids extant progenitor hybridization, evolution and artificial domestication [88,89,90]. Interestingly, many of the detected flowering-related genes were functionally redundant, suggesting that these genes may play an integrated role during evolution and domestication; moreover, the photoperiod pathway may undergo cross-talk with the vernalization and hormone pathways and play a substantial role in regulating cotton flowering. Inversely, most of the flowering-related homologous that were found in *A. thaliana* but not in *G. hirsutum* had a single function, implying that these genes may not participate in the core flowering pathways. Moreover, the genetic localization results show that the flowering mechanism of cotton might be relatively conserved among the allotetraploid species and between the subgenomes. Meanwhile, more than 30% of the light signaling (38.78%), vernalization (30.86%) and aging (47.5%) gene sets were lost. Considering that currently grown cotton varieties evolved and were domesticated from perennial shrub plants in the subtropics [69,89,90], the loss of genes in the photoperiod pathway, vernalization, and aging gene sets may be related to changes in photoperiod and environmental temperature during their northward movement. Notably, all of the floral pathway integrator SOC1 [72,73] and the floral meristem-identity genes *AP1* [2,74], *LFY* [75,76,77,78] and *SVP* [79] are activated to regulate floral primordium initiation, facilitate the transition from vegetative growth to reproductive growth, and accelerate early flowering in SAM during the transition from vegetative growth to reproductive development in *Arabidopsis* [4,73], and all of them were identified in *G. hirsutum*. In addition, a previous study had investigated that GhCAL can form heterodimers with GhAP1/GhAGL6 to regulate their expression and facilitate cotton flowering [70]. GWAS results [69] indicate that, despite the loss of some of the flowering-related genes, the core mode of flowering is evolutionarily conserved in *G. hirsutum* and *A. thaliana* to a certain extent, and the basic light signaling-related flowering pathways are likely relatively conserved. These results indicated that the differentiation fate transition from a vegetative SAM to a reproductive SAM might be a pivotal cell biological characteristic affecting the early flowering of cotton. The flowering mechanisms have been relatively conserved during evolution and domestication, and all of these core factors may also play an indispensable role during floral primordium initiation in cotton SAM, which seems to explain why *G. hirsutum* can flower in temperate areas. Interestingly, combined with the core role of *FT* in the flowering of *Arabidopsis*, rice, and temperate crops [1,2,8], the analysis of gene conserved motifs in *G. hirsutum*, showed that *GhFT* have no conserved motifs with other flowering-related genes that identified in this study, which indicated that *GhFT* homologs in cotton likely also play a core part different with other genes during the evolution, and the function of which might can’t be reproduced by other genes.

Additionally, plants perceive light signaling through their leaves [7] through several photoreceptors, such as *FKF1*/*ZTL*/*LKP2* [35,36,37,91,92], *CRY1/2* [34,42] and *PHYA/B/C/D/E* [38,39,40], and induce florigen, a systemic signal that consists of primarily FT protein and moves through the phloem to SAM [5,31,93]. At the SAM, via reprograming the expression of floral identity genes, such as *AP1*, *SOC1*, *LFY* and *SVP*, FT drives SAM differentiation such that floral primordia are produced [2,4]. In this study, the homologs of *GhCRY1/2*, *GhFKF1/ZTL/LKP2* and *GhPHYA/B/C/D/E* were identified in the *G. hirsutum* genome. Consistently, the identification of GA biosynthesis- and metabolism-related genes *GID1*, *GA20ox1/2*, *GA30x1*, and *GA2ox1/2/3/6/8* [82,83,84] as well as the DELLA family members *GAI*, *RGA, RGL1*, *RGL2* and *RGL3* [85,86], coupled with the changes in hormone contents in the SAM and leaves during the transition of floral primordia in cotton. Furthermore, GA signaling can not only function in leaves by promoting the expression of flowering time integrator genes such as *FT* and *TSF* independently of *CO* and *GI* [27], but also regulates the expression of *SPL* genes in both the leaves and the SAM [27]. All of the findings suggest that hormones, especially GA signals and light signals may participate in the initiation of the floral primordia during the transition from vegetative growth to the reproductive stage.

Furthermore, expression pattern analysis indicates that, in cotton, during evolution and domestication, duplicated genes can undergo nonfunctionalization, neofunctionalization, or subfunctionalization. Genes preferentially expressed in stems and leaves may perceive a light signal in the leaves, transport it through the phloem to the SAM, and stimulate the initiation of floral primordia [4,5,31,93]. Remarkably, selective pressure analysis shows that all Ka/Ks ratio values were lower than 0.200, which indicates that the flowering-related genes for cotton underwent strong purifying selection and that the negative selection had acted against extreme polymorphic variants of flowering-related genes in cotton. Additionally, the sequences of flower development and apical meristem response pathway genes exhibit great sequence diversity, with higher Ka/Ks ratios than those of other flowering pathways between not only the *G. hirsutum* and *A. thaliana* genomes but also the *G. barbadense* and *A. thaliana* genomes, indicating that the cotton flowering-related genes underwent strong purification during evolution, and the same was found between *A. thaliana* and *G. barbadense*, and variation in flower development and apical meristem response pathway genes may contribute to the rapid evolutionary capacity to change the thermal requirement to flowering in *G. hirsutum*.

## 4. Materials and Methods

### 4.1. Data Resources

Details regarding the annotated *A. thaliana* genome were downloaded from the TAIR10 website (http://www.arabidopsis.org/index.jsp, accessed on 10 December 2021). *G. hirsutum*, *G. barbadense,* and *G. arboretum* genomic data and the fragments per kilobase of transcript per million mapped reads (FPKM) of the flowering-related genes for *G. hirsutum* were downloaded from the CottonFGD database (https://cottonfgd.org/about/download.html, accessed on 13 January 2022). The genomic, coding, and protein sequences, and the gene expression of flowering-related genes were downloaded from the CottonFGD database (accessed on 25 January 2022).

### 4.2. Identification of Flowering-Related Gene Homologs in Cotton

Homologous genes were detected by using a combination of similarity- and synteny-based approaches. In the similarity-based approach, the flowering-related genes in *A. thaliana* were used as queries, and the BLASTP program was used to perform searches against *G. hirsutum*, *G. barbadense,* and *G. arboretum* protein sequences with the following conditions: E-value < 1 × 10^−20^, identity > 60%, coverage > 75%, and match length > 70 amino acids.

### 4.3. Chromosomal Localization of Flowering-Related Genes in the Cotton Genome

The locations on the chromosomes or scaffolds indicate the distribution of flowering-related genes, and detailed information on genome localization for the predicted *G. hirsutum*, *G. barbadense* and *G. arboretum* flowering-related genes was obtained from the cotton genome data. A map was constructed using the software of MapChart (MapChart 2.32, which is supported by dr.ir. RE (Roeland) Voorrips in Wageningen, The Netherlands; https://www.wur.nl/en/show/Mapchart.htm, accessed on 5 February 2022) to visualize the putative flowering-related genes on pseudomolecular chromosomes.

### 4.4. Analysis of Multiple Alignments and Phylogenetic, Gene Structure and Motif Recognition for Flowering-Related Genes

Multiple gene protein sequences were aligned by using ClustalW 1.81 software, which is currently maintained at the Conway Institute UCD Dublin by Des Higgins, Fabian Sievers, David Dineen, and Andreas Wilm (Dublin, Germany). A phylogenetic tree was constructed using the MEGA 6.0 program with the maximum likelihood (ML) method (1000 bootstrap replicates) on the basis of full-length protein sequences. 

To visualize the structure of flowering-related genes and illustrate the organization of exons and introns in *G. hirsutum*, Gene Structure Display Server 2.0 (GSDS 2.0, http://gads.cbi.pku.edu.cn/index.php, accessed on 16 February 2022) was used.

Multiple Em for Motif Elicitation (MEME Version 4.11.4, http://meme-suite.org/tools/meme, accessed on 17 February 2022) was used to identify the conserved motifs of the protein sequences of significant flowering-related genes in *G. hirsutum* with default parameters except for the maximum number of motifs, which was set to 25.

### 4.5. Ka/Ks Ratios of Flowering-Related Gene Pairs between A. thaliana and G. hirsutum/G. barbadense

The Ka/ Ks ratios of homologous gene pairs were calculated to investigate the molecular evolution of homologous gene pairs. In the calculations of Ka/Ks, the full-length amino acid sequences of the *G. hirsutum/G. barbadense* and *A. thaliana* flowering-related genes underwent pairwise alignments using MUSCLE. Then, the aligned amino acid sequences were translated into the corresponding nucleotide coding sequences using Perl scripts derived from ParaAT_2.0 software. Lastly, the translated nucleotide coding sequences were used as input files for computing Ka/Ks values by using the Phylogenetic Analysis by Maximum Likelihood (PAML) program (PAML4.9j, http://www.bork.embl.de/pal2nal/index.cgi, accessed on 25 February 2022) with the default parameters. All variable sites of the alignment pairs were used in the calculation of Ka/Ks.

## 5. Conclusions

These findings provide genomic evidence and genomewide information for the investigation of the mechanism of early cotton maturation, and the flowering mechanism of cotton might be relatively conserved among the allotetraploid species and between the subgenomes. The core mode of flowering is also evolutionarily conserved between *G. hirsutum* and *A. thaliana*, and the basic light and hormone-signaling-related flowering pathways are likely relatively conserved. Additionally, the cotton flowering-related genes underwent strong purification during evolution. On the basis of these results, we provide a possible regulatory model of the multiple feedbacks and inputs during the regulation of cotton flowering in the leaves and SAM, which are further systematic and comprehensive flowering regulatory molecular networks of allotetraploid cotton species.

## Data Availability

Not applicable.

## Supplementary Materials

The following supporting information can be downloaded at: https://www.mdpi.com/article/10.3390/ijms23147940/s1.
