# Supplementary material for: Genomewide Identification and Characterization of the Genes Involved in the Flowering of Cotton
Source: Int J Mol Sci. 2022 Jul 19;23(14):7940. doi: 10.3390/ijms23147940 (PMC9323069; doi:10.3390/ijms23147940)
Supplement: Supplementary file 1 [file ijms-23-07940-s001.zip › Supplementary Figures.pdf]

The figure materials for

Genome-wide identification and characterization of the genes involved in the flowering of cotton

This PDF file includes:

Figure. S1 Diverse flowering-related gene sets in *A. thaliana*, *G. hirsutum*, *G. barbadense* and *G. arboreum*.

Figure. S2 Distribution of flowering-related genes on *G. barbadense* chromosomes.

Figure. S3 Functional classification of flowering-related genes.

Figure. S4 Downstream and upstream flowering-related genes.

Figure. S5 Detailed elements of the 25 conserved motifs are shown.

Figure. S6 Detailed elements of the 10 conserved motifs are shown.

Figure. S7 Balance of endogenous hormones during the differentiation of floral meristems.

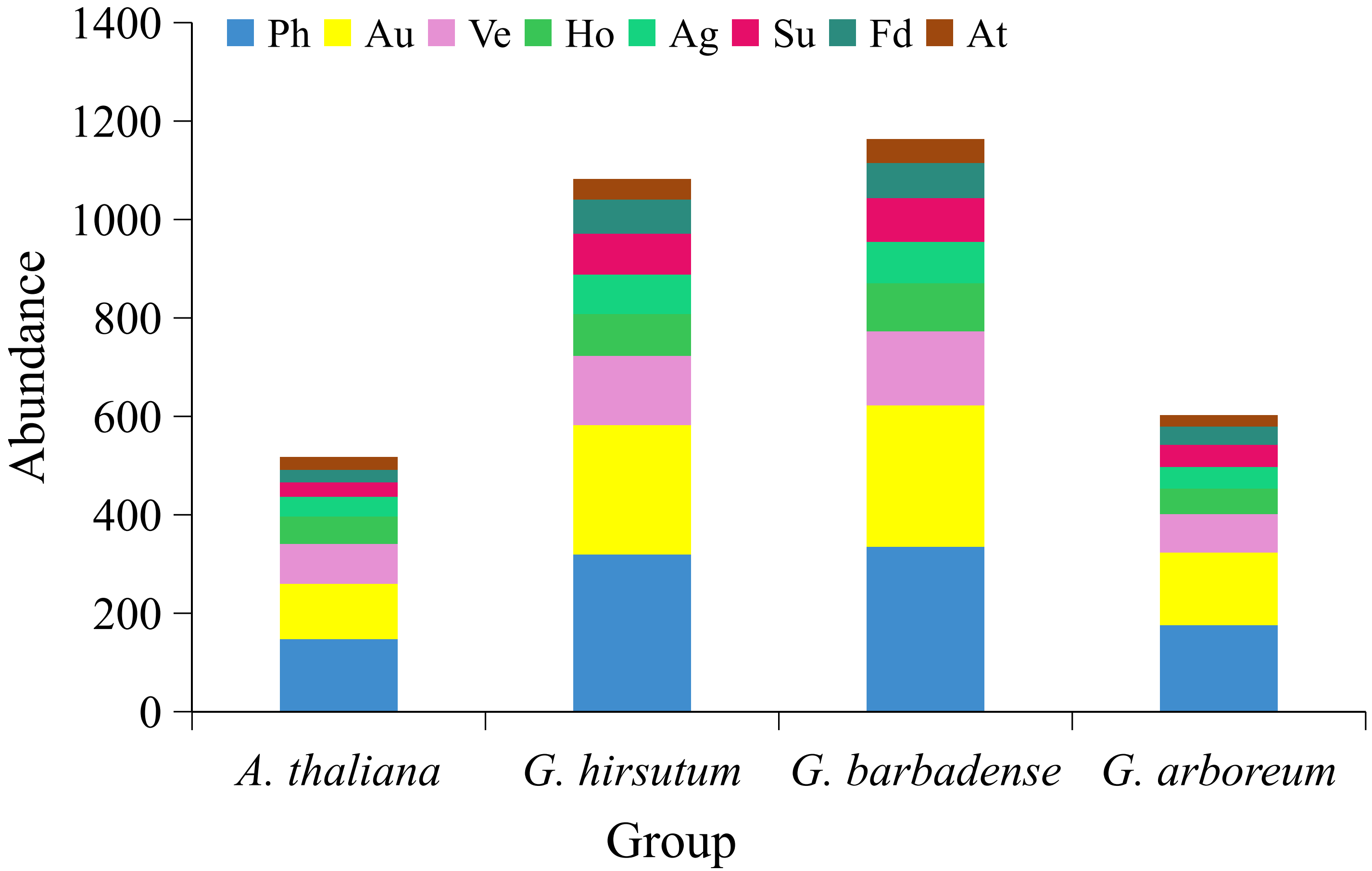

Figure S1 Diverse flowering-related gene sets in *A. thaliana*, *G. hirsutum*, *G. barbadense* and *G. arboreum*. Ph: photoperiodism pathwy; Au: autonomous pathway; Ho: hormones pathway; Ve: vernalization; Ag: aging pathway; Su: sugar signal; Fd: flower development and apical meristem response pathway; At: ambient temperature pathway. At, *A. thaliana*； Ga, *G. arboretum*； Gr, *G. raimondii*； Gh, *G. hirsutum.*

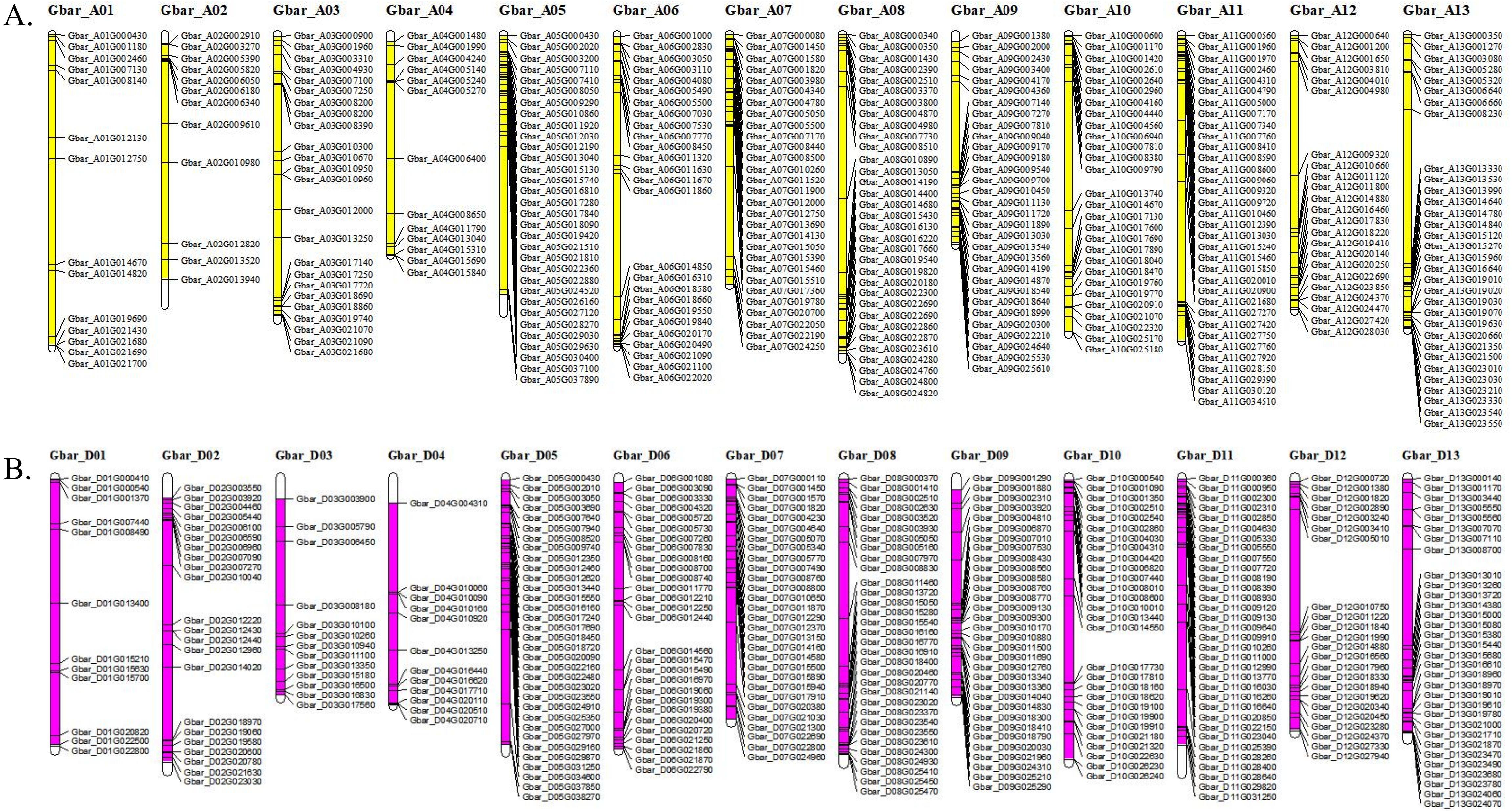

Figure S2 Distribution of flowering-related genes on *Gossypium barbadense* chromosomes.

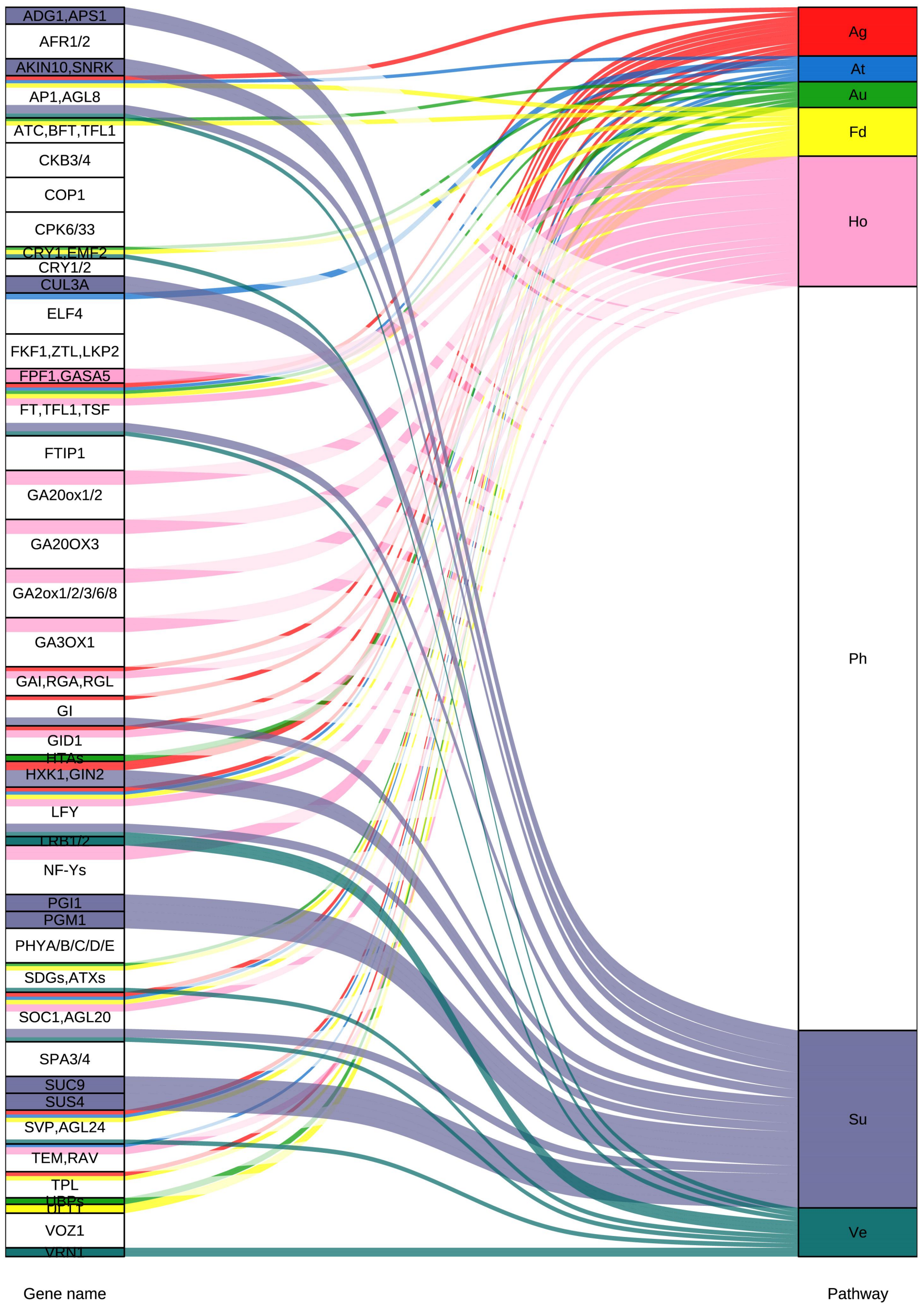

Figure S3 Functional classification of flowering-related genes. The left column represents the flowering gene name, the right column represents the flowering pathways that the flowering genes pertecepate in. Ph: photoperiodism pathway; Au: autonomous pathway; Ho: hormones pathway; Ve: vernalization; Ag: aging pathway; Su: sugar signal; Fd: flower development and apical meristem response pathway; At: ambient temperature pathway.

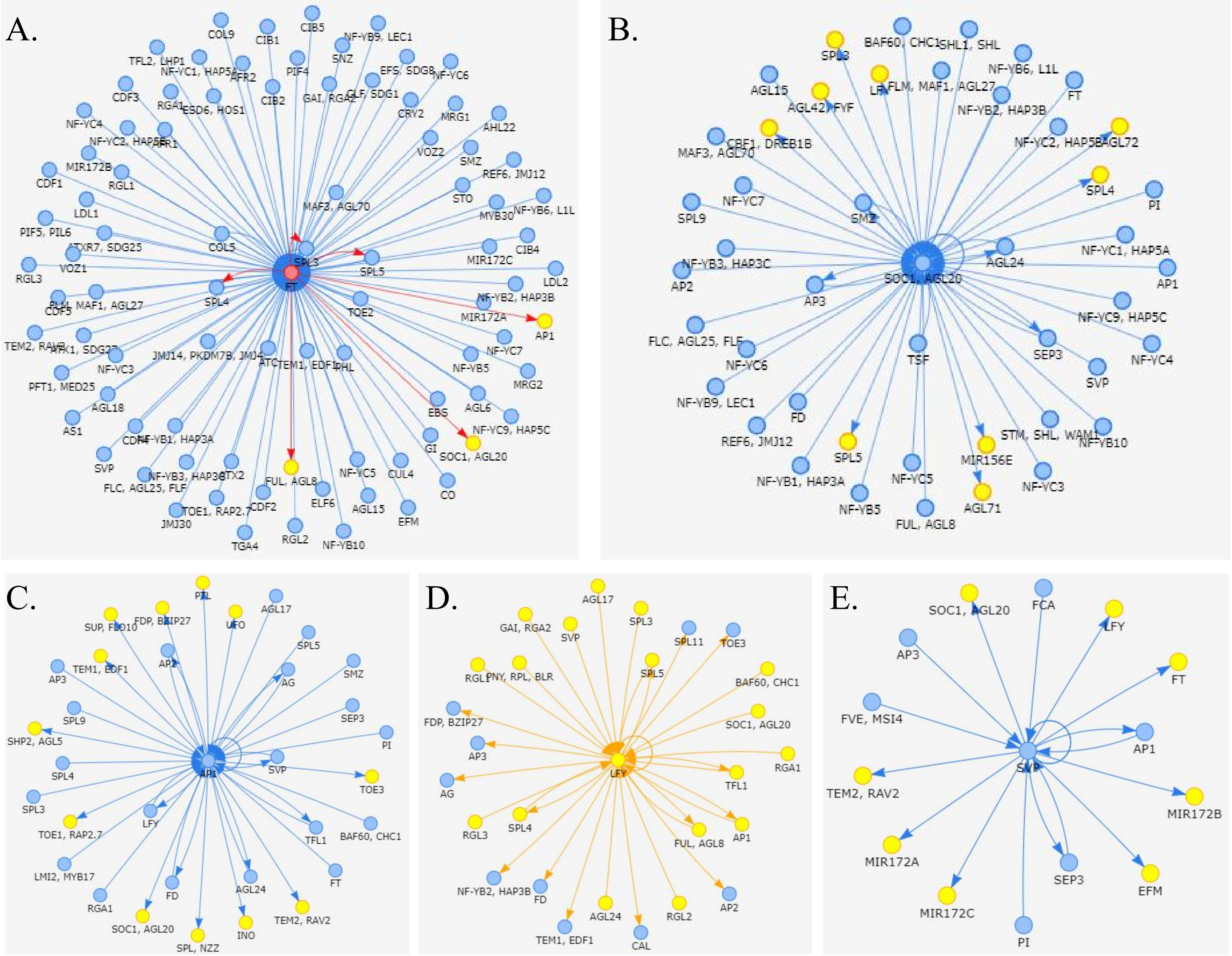

Figure S4 Downstream and upstream flowering-related genes. (A) *FT*. (B) *SOC1*. (C) *LFY*. (D) *AP1*. (E) *SVP*.

The blue circals represent downstream actors; the yellow circals represent upstream actors .

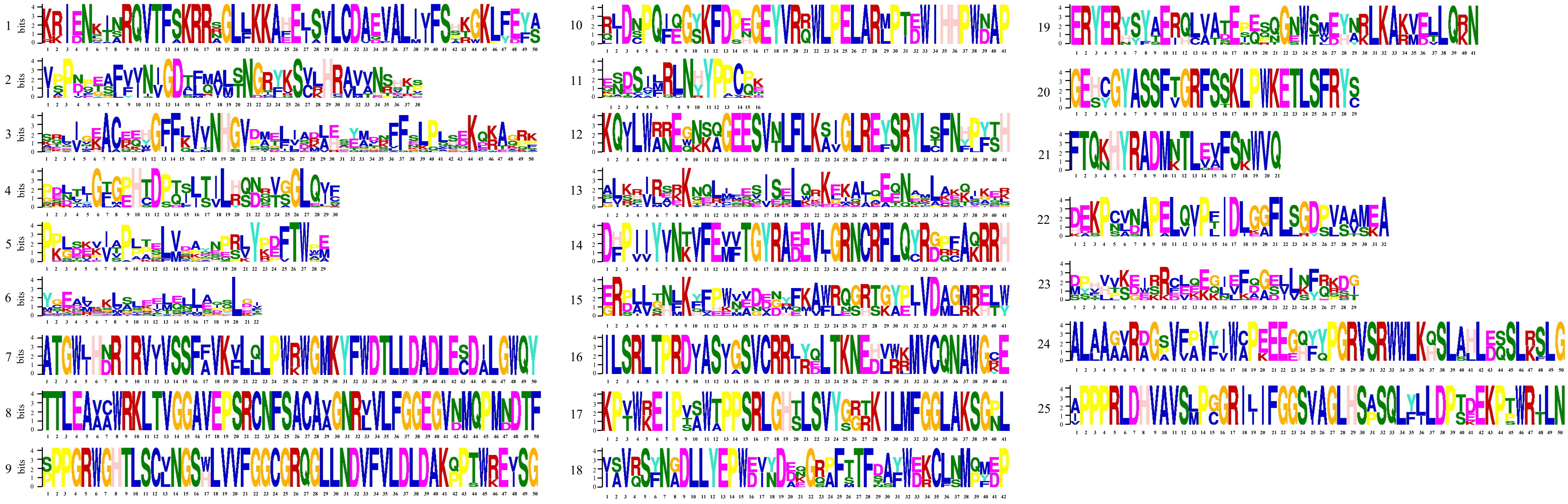

Figure S5 Detailed elements of the 25 conserved motifs are shown.

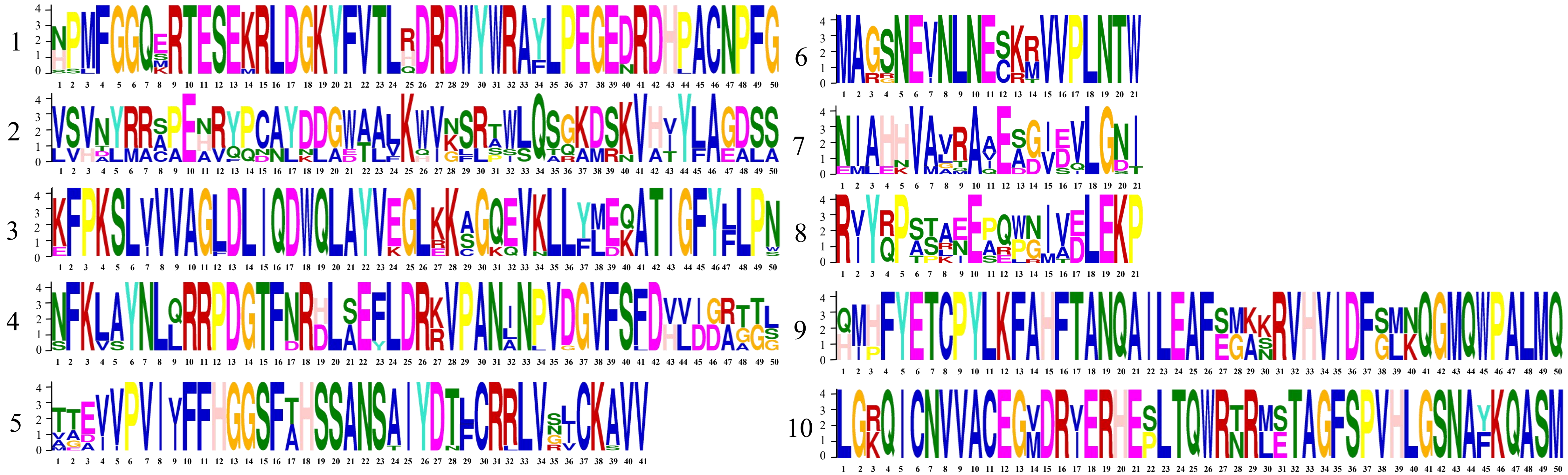

Figure S6 Detailed elements of the 10 conserved motifs are shown.

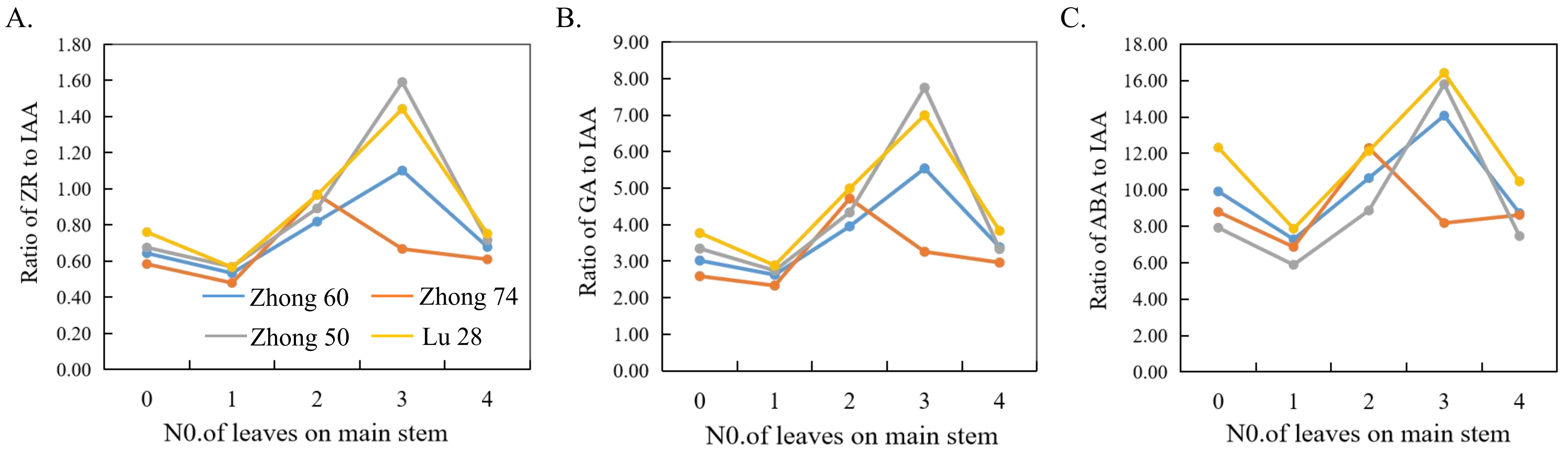

Figure S7 Balance of endogenous hormones during the differentiation of floral meristems. The abscissa represents the development stages for cotton. o, cotyledon period; 1, the first true leaf stage; 2, the second true leaf stage; 3, the third true leaf stage; 4, the four true leaf stage. The vertical axis represents the ratio of hormone content, ratio of ZR to IAA (A); ratio of GA to IAA (B); ratio of ABA to IAA (C). Two early-maturing cultivars: Chinese Cotton Research Institute 50 (CCRI50, also named Zhong50) and Chinese Cotton Research Institute 74 (CCRI74, also named Zhong74); two late-maturing cultivars: Chinese Cotton Research Institute 60 (CCRI60, also named Zhong60) and Luyanmian28 (Lu28).
